# Long‐Term, Stable Alkaline Electrolysis with a Durable Crystalline Polybenzimidazole Membrane

**DOI:** 10.1002/smll.202502810

**Published:** 2025-08-07

**Authors:** Tae Kyung Lee, MinJoong Kim, Hyungkyu Cho, Seungju Lee, Junghwan Kim, Abu Zafar Al Munsur, Byeol‐Nim Lee, Ji Eon Chae, Jonghee Han, Hyun Seo Park, Jong Hyun Jang, Sung Jong Yoo, Sae Yane Peak, So Young Lee, Hyun‐Seok Cho, Soo‐Young Park, Kwang Ho Song, Hyoung‐Juhn Kim

**Affiliations:** ^1^ Hydrogen and Fuel Cell Research Center Korea Institute of Science and Technology (KIST) Hwarang‐ro 14‐gil 5, Seongbuk‐gu Seoul 02792 Republic of Korea; ^2^ Department of Chemical and Biological Engineering Korea University Anam‐ro 145, Seongbuk‐gu Seoul 02841 Republic of Korea; ^3^ Department of Energy Engineering Korea Institute of Energy Research (KIER) Gajeong‐ro 152, Yuseong‐gu Daejeon 34129 Republic of Korea; ^4^ Institute for Hydrogen Energy Korea Institute of Energy Technology (KENTECH) 21 Kentech‐gil Naju Jeonnam 58330 Republic of Korea; ^5^ Department of Mobility Power Research Korea Institute of Machinery & Materials Gajeongbuk‐ro 156, Yuseong‐gu Daejeon 34103 Republic of Korea; ^6^ Materials Architecturing Research Center Korea Institute of Science and Technology (KIST) Hwarang‐ro 14‐gil 5, Seongbuk‐gu Seoul 02792 Republic of Korea; ^7^ Chemical and Biomolecular Engineering Department Sogang University 35 Baekbeom‐ro, Mapo‐gu Seoul 04107 Republic of Korea; ^8^ School of Applied Chemical Engineering Polymeric Nano Materials Laboratory Kyungpook National University Daehak‐ro 80, Buk‐gu Daegu 41566 Republic of Korea

**Keywords:** alkaline electrolysis, biaxial stretching, membrane electrode assembly, non‐PGM catalyst, polybenzimidazole

## Abstract

To overcome the shortcomings of conventional alkaline electrolyzers, polybenzimidazole (PBI) is used to develop novel alkaline electrolysis membranes. In this study, highly crystalline poly(2,2‐*p*‐phenylene‐5,5‐bibenzimidazole) (*p*‐PBI) is synthesized; specifically, the crystallinity of *p*‐PBI is increased during in situ membrane fabrication by introducing an innovative yet simple biaxial stretching step. The biaxially stretched *p*‐PBI (fe‐*p*‐PBI) exhibits high crystallinity, which directly affects the electrolytic cell performance. fe‐*p*‐PBI is evaluated using platinum‐group metal (PGM) and Ni‐based catalysts for alkaline electrolysis under various KOH solution feed conditions at different temperatures. The PGM and Ni‐based cells exhibit current densities of 4.3 and 0.9 A cm^−2^, respectively, at 1.9 V. Further, the electrolysis proceeds without significant damage to the membrane for 900 h at 60 °C. The high crystallinity of fe‐*p*‐PBI contributes to the high performance and long‐term stability of the alkaline electrolyzer, rendering it suitable for use in practical alkaline water electrolysis applications.

## Graphical abstract

This study aims to develop a novel membrane for alkaline electrolysis using poly(2,2‐p‐phenylene‐5,5‐bibenzimidazole) (*p*‐PBI). A convenient technique is employed to induce crystallinity while the membrane is manufactured. Owing to the enhanced crystallinity, the stability of alkaline electrolysis using *p*‐PBI is significantly improved.

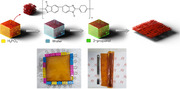

## 1 Introduction

Owing to fossil fuel depletion and climate change, renewable energy, particularly solar and wind power, has attracted considerable attention. However, because these energy sources are not constantly available, their outputs can be non‐uniform. Among the solutions developed to overcome these shortcomings, one solution involves producing green hydrogen using electrolysis techniques powered by surplus renewable energy sources, which is essential to produce grid‐scale intermittent electricity.^[^ ^1^ ^]^ Hydrogen is a versatile raw material and used as most promising energy vector because of its high energy density, interconvertibility to electricity, storage, and simplicity of transportation.^[^ ^2^ ^]^ As it can be used to fuel a variety of hydrogen energy conversion devices, hydrogen is an excellent option to achieve decarbonization of the transportation and industrial sectors.^[^ ^3^ ^]^

Water electrolysis processes are classified into three categories based on the electrolyte, namely, proton exchange membrane (PEM) electrolyzers, alkaline electrolyzers, and solid oxide electrolyzer cells (SOEC). The PEM and alkaline electrolyzers operate at 60–80 °C, whereas the SOEC operates at 600–1000 °C. Among them, PEM electrolyzers are considered most suitable for producing hydrogen using renewable energy owing to their rapid startup, compact design, electric cycling abilities, highly pure hydrogen production, and high current density operation.^[^ ^4^ ^]^ Because PEM electrolyzers require expensive materials such as platinum‐group metal (PGM) catalysts, perflorosulfonic acid (PFSA) polymers, and porous transport layers (PTLs), attention has now turned to alkaline electrolyzers.^[^ ^5^ ^]^ However, conventional alkaline electrolyzers are challenging to operate in the high current density region. In addition, alkaline electrolysis is susceptible to heavy‐duty cycle operation or startup/shutdown because it requires a liquid electrolyte and porous diaphragm. Owing to the structural limitations of the diaphragm, gas crossover occurs through the pores during high pressure and differential pressure operations, which negatively affects the hydrogen production efficiency and stability.

To overcome the limitations of PEM and conventional alkaline electrolyzers, anion exchange membrane (AEM) electrolyzers have been developed.^[^ ^6^, ^7^ ^]^ An AEM electrolyzer contains a dense solid membrane similar to those of a PEM electrolyzer rather than the porous diaphragm of alkaline electrolyzers. In addition, because AEM electrolyzers are operated under alkaline conditions, non‐precious metal catalysts can be used instead of the PGM catalysts required for PEM electrolyzers.^[^ ^8^, ^9^, ^10^, ^11^, ^12^, ^13^ ^]^ Recently, commercial membranes from Tokuyama and Fumatech have been used to construct AEM electrolyzers^[^ ^14^, ^15^, ^16^, ^17^, ^18^, ^19^ ^]^ and new AEM electrolyte membrane materials have been studied for electrolyzer operation in KOH solution or pure water.^[^ ^20^, ^21^, ^22^, ^23^, ^24^ ^]^ However, these polymers lack long‐term durability, and many studies only focused on early‐stage performance because of the limited alkaline stability. Nevertheless, after extensive research, membranes with excellent long‐term stability have been developed. Recently, Motealleh et al. demonstrated good electrolytic performance using a Sustainion AEM from Dioxide Materials.^[^ ^25^ ^]^ A membrane electrode assembly (MEA) with a Sustainion AEM exhibited an excellent performance durability of ≈1 µV h^−1^ over 10 000 h. In addition, Chen et al. reported high‐performance poly(fluorenyl co‐aryl piperidinium)‐based water electrolysis that exhibited 1000 h of long‐term durability.^[^ ^26^ ^]^ More recently, Jang et al. demonstrated a three‐cell AEM electrolyzer stack with a polycarbazole‐based AEM and non‐PGM catalysts.^[^ ^27^ ^]^ The stack was operated for 2000 h in 0.1 m KOH at 45 °C.

Polybenzimidazole (PBI) derivatives have been used for alkaline electrolyzers.^[^ ^28^, ^29^, ^30^, ^31^ ^]^ PBIs are stable materials that exhibit excellent alkali resistance and are therefore suitable for use in alkaline electrolyzers. In particular, Kraglund et al. developed an alkaline electrolyzer that exhibited a very high performance (1700 mA cm^−2^ at 1.8 V) using poly(2,2‐*m*‐phenylene‐5,5‐bibenzimidazole) (*m*‐PBI) via an ion solvation process.^[^ ^31^ ^]^ However, the long‐term stability of PBI‐based AEM electrolyzers is poor. Previous studies noted that mechanical failure of the PBI membrane, such as holes and tears in the membrane, occurred within 300 h of operation.^[^ ^32^ ^]^ Although *m*‐PBI is chemically stable, it is thought that the physical properties of the polymer deteriorate because it absorbs a large amount of KOH solution.

In this study, poly(2,2‐*p*‐phenylene‐5,5‐bibenzimidazole) (*p*‐PBI) was synthesized in polyphosphoric acid, and a membrane was fabricated from the polymerization mixture. The acid‐doped membrane was washed with water and isopropyl alcohol (IPA), respectively. The membrane was then biaxially stretched to improve crystallinity. The 2D wide‐angle X‐ray scattering (WAXS) patterns of the biaxially stretched *p*‐PBI (fe‐*p*‐PBI) confirmed the high crystallinity of the polymer membrane, which improved the long‐term durability of PBI membrane‐based alkaline electrolysis. The fe‐*p*‐PBI membrane was evaluated under alkaline conditions (15 wt.% KOH solution), and electrolysis was performed for 900 h. When alkaline water electrolysis operated under strong base conditions, the advantage is that water electrolysis can be operated using an inexpensive catalyst compared to weak base conditions of 1 m or less. The crystallinity of a polymer affects its alkali resistance: the higher the crystallinity, the better the alkali resistance. To date, no studies have focused on the durability of polymer membranes in alkaline electrolyzers from a polymer crystallinity perspective. This is the first study reporting an easily fabricated polymer with superior crystallinity for long‐term durability. The developed membranes show significant potential and can be integrated into existing alkaline water electrolysis systems to increase the single cell performance and durability.

However, the crystallinity of fe‐*p*‐PBI was not maintained during the entire electrolysis operation, and an increase in hydrogen permeability through the fe‐*p*‐PBI membrane was observed. Although fe‐*p*‐PBI remains stable in high concentration KOH solution, the stability does not appear to be secured in electrochemical reactions. In this paper, we report the simple fabrication of crystalline PBI and its durability in alkaline electrolysis operation. In particular, we describe the relationship between hydrogen permeability and water electrolysis performance.

## 2 Results and Discussion

### 2.1 Electrolyte Membrane Fabrication and Characterization

**Figure 1:**
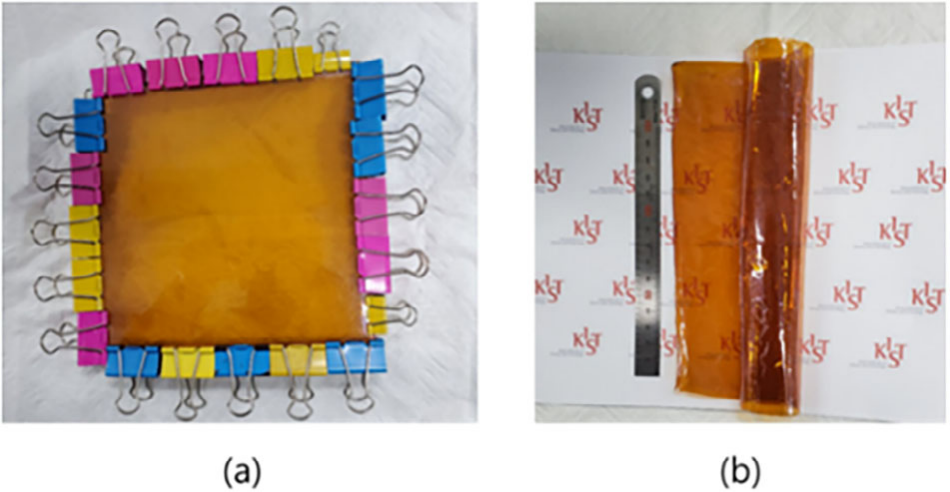
Fabrication of fe‐*p*‐PBI a) immediately after fixing the membrane to the glass and b) after evaporation of isopropanol.

*p*‐PBI and *m*‐PBI were synthesized using our previously published methods.^[^ ^33^, ^34^, ^35^ ^]^ The structures of the PBI derivatives are shown in Figure S1 (Supporting Information). Immediately after synthesizing the PBI derivatives, the polymer mixture was cast on a glass plate to obtain the membrane (in situ membrane fabrication). The membrane was quenched in water to detach it from the glass plate and remove the polymerization solvent. The membrane was then placed in IPA bath. After being removed from the bath, the membrane was placed on a glass plate, with its four corners fixed to the glass plate with double clips (**Figure** **1**a). After the IPA was evaporated, a biaxially extended membrane (fe‐*p*‐PBI) was formed. During the evaporation process, the PBI membrane became thin and dense with a crystalline morphology, as shown in Figure S2 (Supporting Information). The membrane (pristine‐*p*‐PBI) that was directly dried without applying the biaxial stretching process was severely contracted and deformed, resulting in a form that was unsuitable as an electrolyte membrane. In contrast, an fe‐*m*‐PBI electrolyte membrane could not be produced using this process because the membrane broke during the production process (Figure S3, Supporting Information). This is thought to occur because *m*‐PBI has a lower inherent crystallinity and mechanical strength than *p*‐PBI. The 2D WAXS analyses of the *p*‐PBI and *m*‐PBI fiber crystallinities (Figure S4, Supporting Information) revealed that the morphology of the *p*‐PBI fiber was significantly more crystalline than that of the *m*‐PBI fiber. The chemical structures of their respective monomers suggest that *m*‐PBI will exhibit a curved structure, whereas the linear structure of *p*‐PBI imparts higher crystallinity and mechanical strength. Owing to these characteristics, *p*‐PBI can easily produce a biaxially stretched membrane, whereas *m*‐PBI cannot withstand the biaxial stretching process. Consequently, we used solution casting to prepare an *m*‐PBI (sc‐*m*‐PBI) membrane from a dimethylacetamide (DMAc) solution of 2 wt.% *m*‐PBI for comparison.

**Figure 2:**
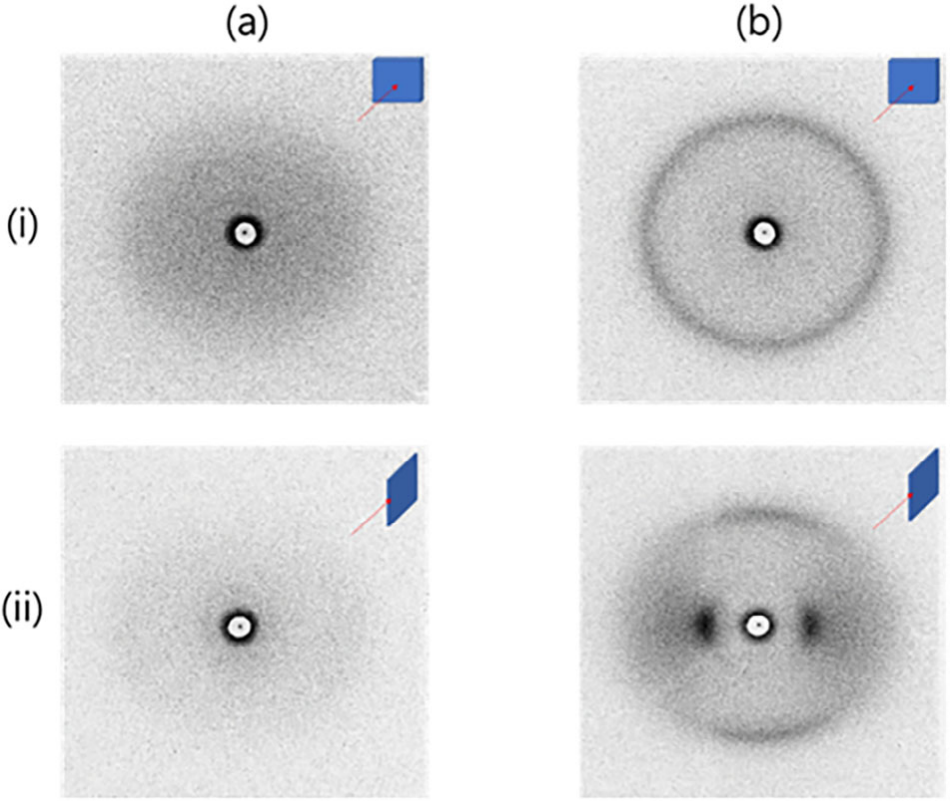
WAXS patterns of the a) sc‐*m*‐PBI and b) fe‐*p*‐PBI membranes under X‐ray irradiation (i) perpendicular and (ii) parallel to the membrane surface; insets indicate the X‐ray beam direction.

The crystal orientations in the fe‐*p*‐PBI and sc‐*m*‐PBI membranes were determined using 2D WAXS patterns in which the X‐ray beam was either parallel or perpendicular to the membrane surface (**Figure** **2**). The 2D WAXS patterns of the sc‐*m*‐PBI membrane showed minimal scattering under both parallel and perpendicular X‐ray irradiation owing to its amorphous structure (Figure 2a), whereas the patterns of the fe‐*p*‐PBI (Figure 2b) membranes exhibited two distinct scatterings at Q = 0.89 Å^−1^ (d‐spacing = 7.08 Å) and 1.85 Å^−1^ (d‐spacing = 3.40 Å) under different orientations depending on the direction of the X‐ray beam. When X‐ray beam was perpendicular to the membrane surface (Figure 2b‐i), the scattering at Q = 1.85 Å^−1^ was isotropic (circular) and the scattering at Q = 0.89 Å^−1^ was almost invisible. When the X‐ray beam was parallel to the membrane surface (Figure 2b‐ii), the scatterings at Q = 0.89 and 1.85 Å^−1^ were located along the equatorial and meridional directions, respectively. The scattering at Q = 0.89 Å^−1^ was attributed to the packing between the aromatic rings; the equatorial location indicated that the aromatic rings were oriented parallel to the membrane surface, which prohibited the Ewald scattering condition when the X‐ray beam was perpendicular to the membrane surface (Figure 2a‐i). The scattering at Q = 1.85 Å^−1^ was attributed to the repeating units along the chain direction. When the X‐ray beam was parallel to the membrane surface, the meridional location indicated that the PBI chains were oriented parallel to the membrane surface. Thus, the fe‐*p*‐PBI membrane exhibited a uniaxial planar orientation that formed spontaneously during the membrane preparation.

**Figure 3:**
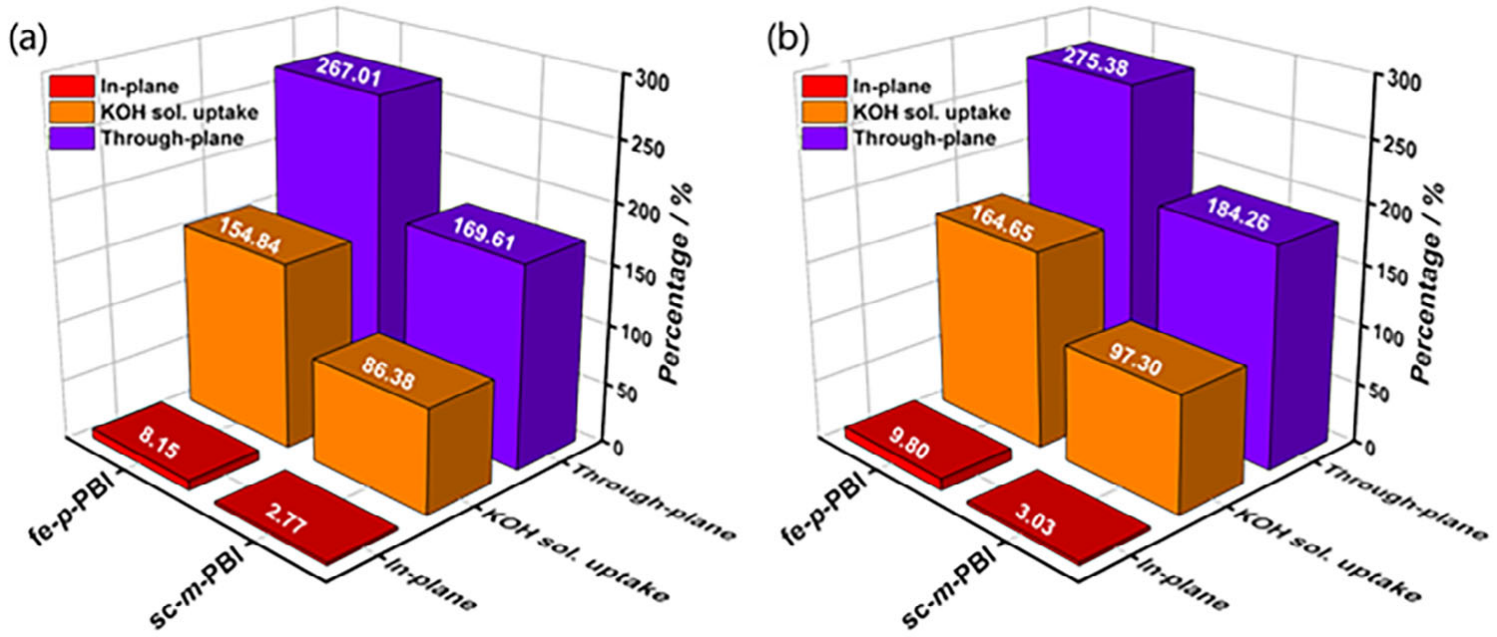
KOH solution (20 wt.% aqueous KOH) swelling, and in‐plane and through‐plane swelling of fe‐*p*‐PBI and sc‐*m*‐PBI at a) 30 °C and b) 80 °C.

**Figure** **3** presents the KOH solution swelling of the fe‐*p*‐PBI and sc‐*m*‐PBI membranes at 30 and 80 °C. KOH solution swelling occurred primarily in the through‐plane direction of the electrolyte membrane and was negligible in the in‐plane direction. In general, highly crystalline polymers swell less than amorphous polymers of similar structure due to their compact packing. Nevertheless, the KOH solution swelling of fe‐*p*‐PBI was higher than that of sc‐*m*‐PBI. We hypothesized that the ordered orientation of the fe‐*p*‐PBI crystals and the plasticizing effect of the absorbed KOH solution enabled a larger biaxial expansion of the polymer membrane, resulting in greater absorption of the KOH solution.

*p*‐PBI itself is not hydroxyl ion conductive. *p*‐PBI absorbs KOH solution and the latter acts as an electrolyte. Therefore, the conductivity of electrolysis cell increases with the concentration of the KOH solution. fe‐*p*‐PBI membranes absorbing different concentrations of KOH solution were placed in an environmental chamber, and their conductivities were measured at 60 and 80 °C. As shown in Table S1 (Supporting Information), the conductivity ranged from 67.7 to 103.7 S cm^−1^, showing increase as the membrane was treated with increased concentrations. The membrane also showed higher conductivity at higher temperatures. The conductivity of fe‐*p*‐PBI exhibited excellent agreement with the performance of the electrolysis cell described later.

### 2.2 Water Electrolysis Using PBI Membranes

Before the fe‐*p*‐PBI was used as the membrane in an electrolyzer, its stability in KOH solution was tested. Table S2 (Supporting Information) shows that the mechanical properties of the membrane remained unchanged after immersion in 1 m KOH solution at 80 °C for 500 h. Notably, there was no significant change in the thickness and area of the fe‐*p*‐PBI membrane before and after measurement. Based on this, the chemical stability of fe‐*p*‐PBI was confirmed and its actual performance was evaluated by fabricating a water electrolysis single cell.

Additionally, the fe‐*p*‐PBI membrane was soaked in harsher conditions: KOH 6 m, 80 °C for 250 h. At this time, weight of the membrane decreased by 3%. We used 2D WAXS patterns to investigate the changes in polymer morphology for fe‐*p*‐PBI membrane in a strongly alkaline condition. Figure S5 (Supporting Information) shows the 1D data scanned along the 2θ direction from the 2D WAXS patterns of the fe‐*p*‐PBI and fe‐*p*‐PBI after treatment in the KOH solution. Two peaks are mainly visible at Q = 0.89 and 1.85 Å^−1^ corresponding to the planar packing between the aromatic rings and the repeating aromatic units along the chain direction, respectively, as mentioned previously. The peak at Q = 0.89 Å^−1^ is invisible (low intensity) when the planar orientation is high due to Ewald sphere diffraction. This weak peak is enhanced after KOH treatment, indicating that the planar orientation is lost after the strong alkali treatment. The peak at Q = 1.85 Å^−1^ also broadens after the KOH treatment, implying that the crystalline may break into the smaller one owing to the KOH treatment. This phenomenon is believed to occur because the KOH solution penetrates into the main chain of fe‐*p*‐PBI and breaks its high crystallinity.

**Figure 4:**
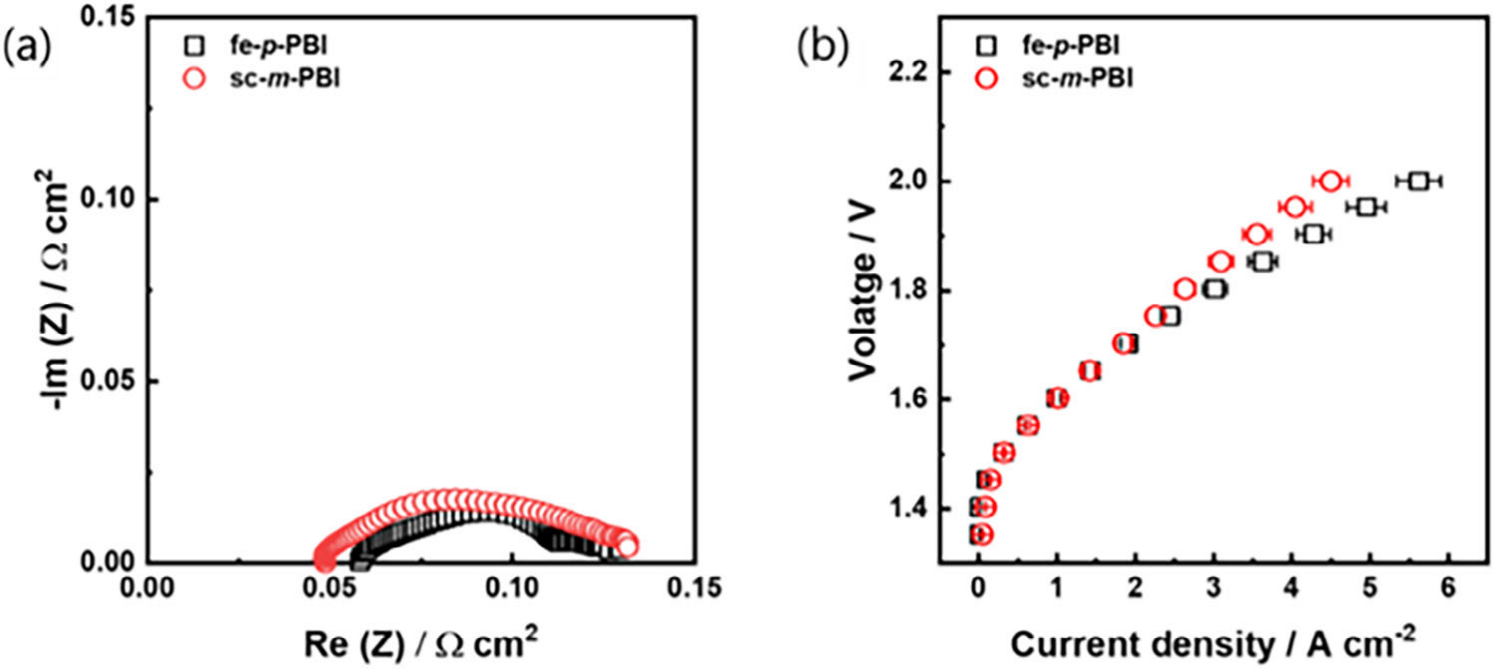
Electrolysis performance of fe‐*p*‐PBI and sc‐*m*‐PBI. (a) Nyquist plots at 1.6 V. (b) Electrolyzer polarization curves (membrane thickness: 30 µm; KOH feed concentration: 20 wt.%; temperature: 80 °C; anode catalyst: IrO_2_ (2 mg_Ir_ cm^−2^) on stainless steel fiber paper; cathode catalyst: Pt/C (1 mg_Pt_ cm^−2^) on Ni fiber paper). Error bars show the variation between the three cells.

MEAs containing fe‐*p*‐PBI or sc‐*m*‐PBI membranes, precious metal catalysts such as Pt/C (cathode) and IrO_2_ (anode), and Nafion dispersion (catalyst binder) were fabricated and evaluated. The configuration of the water electrolyzer, bipolar plates, and single cell are shown in Figure S6 (Supporting Information). Before the MEA test, scanning electron microscope (SEM) analysis and energy dispersive X‐Ray spectrometer (EDS) mapping were performed to confirm that catalysts were uniformly distributed without agglomeration (Figures S7–S10, Supporting Information). A direct comparison of the fe‐*p*‐PBI and sc‐*m*‐PBI membrane properties may not be appropriate owing to their different fabrication procedures. As previously described, *m*‐PBI cannot withstand biaxial stretching owing to its low mechanical strength, and *p*‐PBI has limited solubility in general organic solvents, making it challenging to fabricate an electrolyte membrane using the solution casting method. Therefore, we evaluated the electrolysis performance using the fe‐*p*‐PBI and sc‐*m*‐PBI membranes. The performance of the fe‐*p*‐PBI and sc‐*m*‐PBI cells was similar, despite the higher KOH solution swelling of fe‐*p*‐PBI compared to that of sc‐*m*‐PBI (**Figure** **4**). In addition, the Nyquist plots of the two membrane‐systems were similar. Based on these results, KOH solution swelling does not directly affect the electrolytic cell performance. The electrolysis performance appeared to be similar after the membranes had adsorbed a certain amount of KOH solution; however, further studies are required to monitor the variations in electrolysis performance based on the KOH solution concentration and operating temperature.

**Figure 5:**
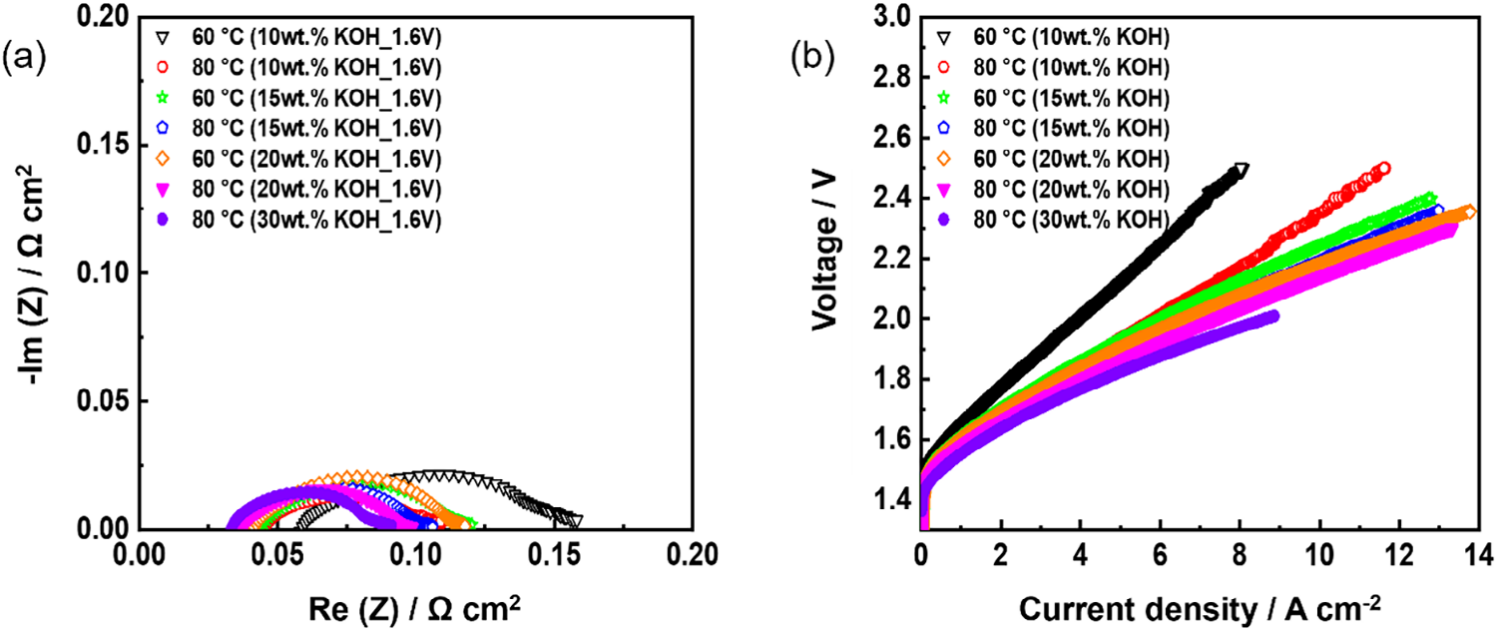
Electrolysis performance of fe‐*p*‐PBI. a) Nyquist plots at 1.6 V. b) Electrolyzer polarization curves (membrane thickness: 30 µm; KOH feed concentration: 10–30 wt.%; temperature: 60, 80 °C; anode catalyst: IrO_2_ (2 mg_Ir_ cm^−2^) on stainless‐steel fiber paper; cathode catalyst: Pt/C (1 mg_Pt_ cm^−2^) on Ni fiber paper.

We evaluated the performance of the cell according to the KOH concentration and temperature change (**Figure** **5**). As expected, the performance of the cell improves with increasing temperature and KOH concentration. The conductivity increases with concentration of the KOH solution, and consequently, the cell performance also improves. As shown in Figure 5a, all resistances are reduced at high KOH feed concentration and operating temperature.

The sc‐*m*‐PBI cell was only stable for 250–300 h, as previously reported by Kraglund et al.^[^ ^31^ ^]^ Although the physical properties of the PBI are strong against bases, its mechanical properties are greatly reduced when it absorbs KOH solution.^[^ ^32^ ^]^ We also confirmed that several sections of the sc‐*m*‐PBI membrane were torn. To overcome these shortcomings of sc‐*m*‐PBI, we attempted to improve its mechanical strength by imparting high crystallinity to PBI. fe‐*p*‐PBI was prepared with the expectation that its long‐term stability would increase due to its high crystallinity, even if it absorbed a large amount of KOH solution.

**Figure 6:**
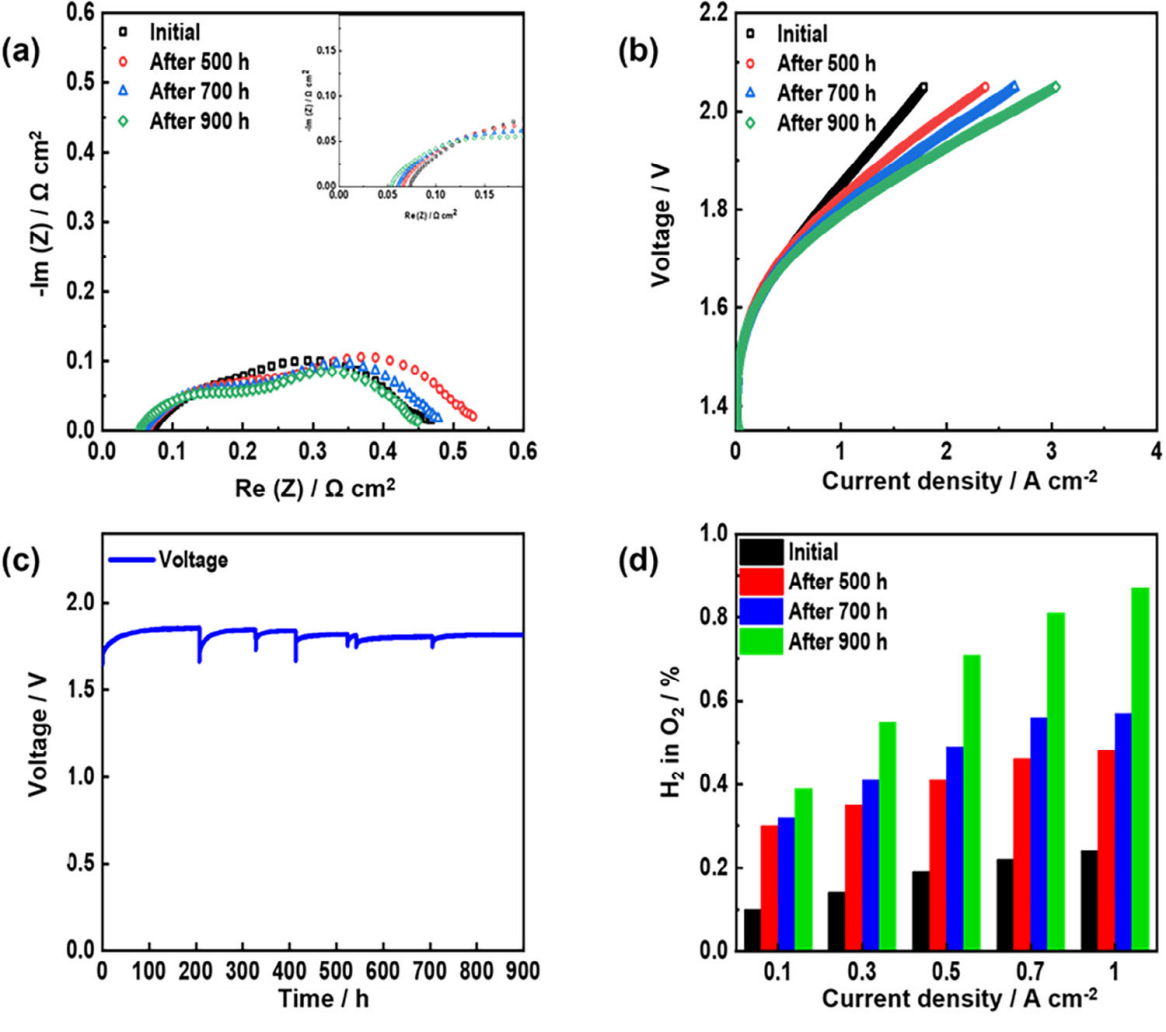
Long‐term stability of fe‐*p*‐PBI operating at constant current density of 0.7 A cm^−2^. a) Nyquist plots at 1.6 V. b) Electrolyzer polarization curves (membrane thickness: 50 µm; KOH feed concentration: 15 wt.%; temperature: 60 °C; anode catalyst: IrO_2_ (2 mg_Ir_ cm^−2^) on stainless steel fiber paper; cathode catalyst: Pt/C (1 mg_Pt_ cm^−2^) on Ni fiber paper). c) Voltage change during 900 h of operation. d) Hydrogen volume percent in oxygen at the anode.

During the long‐term durability test of the fe‐*p*‐PBI, the cell voltage was recorded at a current density of 0.7 A cm^−2^ at 60 °C (**Figure** **6**). The Nyquist plot shows that the ohmic resistance gradually decreased during the 900 h of operation (Figure 6a). As shown in Figure 6b, the cell performance was improved during the long‐term operation. The hydrogen level in oxygen at the anode was measured as a function of current density (Figure 6d). After 900 h of operation, the volume percentage of hydrogen in anode oxygen reached 0.9%, indicating a change in the state of the fe‐*p*‐PBI membrane, which was confirmed using the WAXS pattern of fe‐*p*‐PBI.

**Figure 7:**
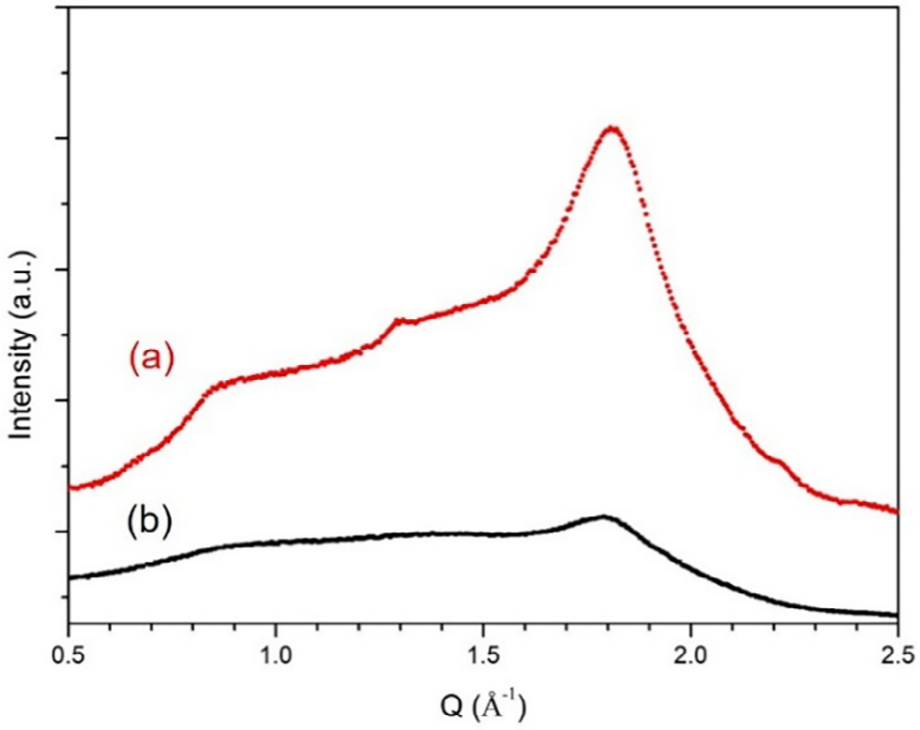
WAXS diffractograms of a) fe‐*p*‐PBI kept in 6 m KOH solution at 80 °C for 250 h and b) fe‐*p*‐PBI operated in the electrochemical cell for 500 h.

The fe‐*p*‐PBI is quite stable in highly concentrated KOH solution. However, during electrolysis operation, fe‐*p*‐PBI does not maintain its initial crystalline structure. We analyzed WAXS pattern of the fe‐*p*‐PBI that was operated electrochemically for 500 h. Unlike the WAXS pattern of the fe‐*p*‐PBI kept in 6 m KOH solution at 80 °C for 250 h, that of the fe‐*p*‐PBI operated in the electrolysis cell showed a reduction in crystal peaks at Q = 0.89 and 1.85 Å^−1^, indicating that crystallinity is considerably reduced by the electrolysis cell operation (**Figure** **7**). In water electrolysis, electrochemical reactions occur that are much more complex and violent than typical chemical reactions. We believe that the crystallinity of the polymer is destroyed during anion exchange through the KOH solution within the fe‐*p*‐PBI membrane.

Damage to the fuel cell electrolyte membranes can be easily recognized as a drop in open circuit potential (OCP) in the polarization curve. In the case of fuel cells, membrane damage can be easily detected even during long‐term constant current operation. However, in water electrolysis, unlike fuel cells, membrane damage due to voltage changes in the polarization curve and constant current operation is difficult to confirm. In our case, the hydrogen sensor detected an increase in the permeation of hydrogen from the cathode to the anode due to damage to the membrane, but it did not appear to reduce the performance of water electrolysis. To confirm the stability of the membrane for water electrolysis, it is considered necessary to measure hydrogen permeability during electrolysis in addition to evaluating cell performance.

The SEM image of the membrane after the durability test is shown in Figure S11 (Supporting Information). The results of the SEM analysis showed that the membrane maintained a dense morphology similar to that of the membrane before the durability measurement. However, SEM images show the phenomenon of membrane thinning. The decrease in resistance, improvement in cell performance, and increase in hydrogen permeability are explained by the membrane thinning.

**Figure 8:**
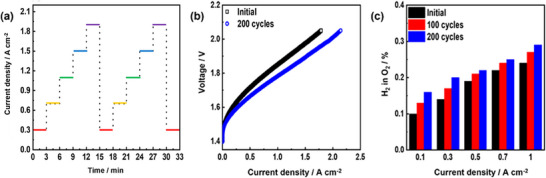
Electrolysis performance of fe‐*p*‐PBI after 200 cycle AST operation. a) Cycle scenario for accelerated life test. b) Electrolyzer polarization curves (membrane thickness: 50 µm; KOH feed concentration: 15 wt.%; temperature: 60 °C; anode catalyst: IrO_2_ (2 mg_Ir_ cm^−2^) on stainless steel fiber paper; cathode catalyst: Pt/C (1 mg_Pt_ cm^−2^) on Ni fiber paper). (c) Hydrogen volume percent in oxygen at the anode resulting from the AST test).

Accelerated stress test (AST) involves dynamic durability, which accelerates degradation by employing the electrolytic cell in stressful operating conditions. Chronopotentiometry was used to assess the cell's AST by applying five constant‐current steps from 0.3 to 1.9 A cm^−2^ (**Figure** **8**a). Each step lasted 3 min, and this was performed for 200 cycles. After AST, the cell performance improved. This phenomenon is similar to the long‐term operation at constant current density. The hydrogen level in oxygen at the anode was measured as a function of current density during the AST. The hydrogen permeability increased significantly with cycling operation. It took 50 h to complete 200 cycles. The hydrogen permeability after 50 h was similar to the permeability after 500 h of constant current operation. This result indicates that the fe‐*p*‐PBI membrane is more seriously damaged during cycling operation.

Ni‐Fe layered double hydroxide (Ni‐Fe LDH) is regarded as an efficient OER catalyst in alkaline water electrolysis.^[^ ^36^, ^37^, ^38^ ^]^ It consists of positively charged layers in which divalent cations (Ni^2+^) are substituted with trivalent cation (Fe^3+^). To balance the charges, anions are intercalated between the positively charged layers by expanding the interlayer distance. This unique open‐layered structure facilitates fast OER kinetics. We prepared a Ni‐Fe LDH anode via a growth method based on the controlled oxygen corrosion of an Fe foam in Ni solution.^[^ ^39^ ^]^ The Ni‐Fe LDH layer was grown on the Fe foam by precipitating the corroded Fe ions and Ni ions from the solution.

In addition, chemically leached porous Ni‐Al catalysts with an optimal chemical state for Ni^2+^/Ni^0^ are expected to replace Pt‐based catalysts for the HER.^[^ ^40^, ^41^ ^]^ We prepared a Raney‐type Ni cathode via the physical vapor deposition (PVD) of Al on a Ni foam with subsequent heat‐treatment (600 °C) and alkaline leaching processes. The gamma‐phase Ni‐Al alloy that was formed during the heat‐treatment of the Al‐coated Ni foam was leached with 20 wt.% KOH solution, which resulted in a porous Raney‐type Ni structure.^[^ ^42^ ^]^

**Figure 9:**
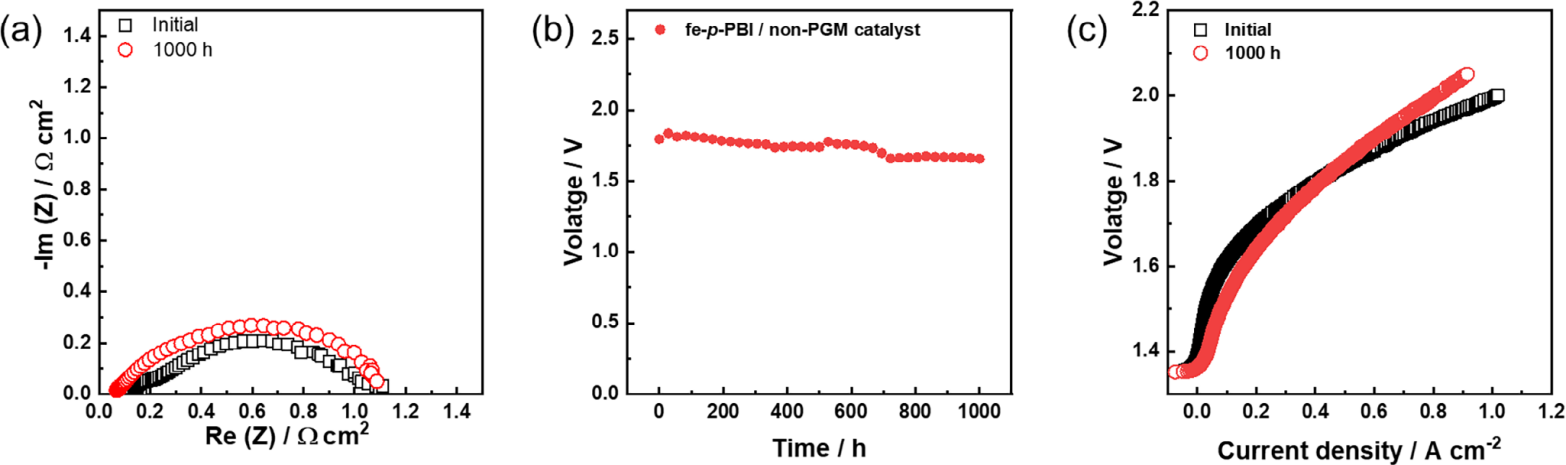
Long‐term stability of single cell operation at a constant current density of 0.5 A cm^−2^ using fe‐p‐PBI with non‐PGM catalysts. a) Nyquist plots (1.6 V) of initial and 1000 h operation. b) Polarization curves of initial and 1000 h operation. c) Long‐term stability during 1000 h operation of fe‐p‐PBI membrane. Membrane thickness: 30 µm; KOH feed concentration: 20 wt.%; temperature: 80 °C; anode catalyst: Ni‐Fe LDH; cathode catalyst: Ni‐Al.

SEM analysis and EDS mapping was performed to confirm the shapes of Ni‐Fe LDH and Ni‐Al electrodes. Both catalysts were confirmed to be well attached on the substrate, and uniformly distributed without agglomeration (Figures S12–S15, Supporting Information). Alkaline electrolysis with the fe‐*p*‐PBI membrane was performed using the Ni‐based electrodes described above. **Figure** **9** shows the performance of the fe‐*p*‐PBI cell using a 20 wt.% KOH feed solution at 80 °C. The performance of the fe‐*p*‐PBI cell with Ni‐based electrodes was lower than that of the precious metal catalyst cell. Figure S16 (Supporting Information) shows the Nyquist plots and polarization curves comparing the performance of alkaline water electrolysis using precious metal and non‐noble metal catalysts. The low performance using non‐PGM catalysts is attributed to the low activity of the catalysts. When non‐precious metals are used in alkaline water electrolysis, electrodes are manufactured in the form of coating on a PTL without using a binder. In this case, since the PTL is a porous material, the contact area of the PTL with the membrane may change depending on conditions such as thickness, shape, and porosity of the PTL. It is thought that as the contact area between the membrane and the electrode decreases and the interfacial resistance between them increases, thereby deteriorating the performance of the electrolyzer using non‐precious metals.

We conducted EIS measurements for PGM and non‐PGM based electrodes at 0 V (Figure S17, Supporting Information). The stainless‐steel fiber and Ni fiber papers were used for the PTL of PGM electrodes. Fe and Ni foams were used for the PTL of non‐PGM electrodes. As shown in Figure S17 (Supporting Information), the ohmic resistance of non‐PGM‐based electrodes is greater than that of PGM‐based electrodes. This is likely due to differences in the PTL; the non‐PGM PTL, such as foam, is thicker and has higher tortuosity compared with the fiber paper used for the PGM electrode. Fiber paper is typically 300–650 µm thick, whereas the thickness of the foam exceeds 1.5 mm. The greater thickness and tortuosity of the foam can hinder effective reactant/gas product transport, leading to increased ohmic resistance (2nd semi‐circle), as shown in Figure S16a (Supporting Information).

Porous electrode structures and surface heterogeneities can introduce non‐idealities in charge transfer processes, influencing the formation and behavior of the electrochemical double layer. These deviations often manifest as an inclined linear region in Nyquist plots. Non‐PGM electrodes, characterized by greater structural heterogeneity and irregularities compared with PGM electrodes, are more likely to exhibit restricted electron transport within the electrode, PTL, and interaction with the electrolyte, contributing to the observed slope. Furthermore, gas entrapment within the PTL or at the electrode‐electrolyte interface can obstruct reactant transport, thereby increasing mass transport resistance. The increased thickness of PTL of non‐PGM electrodes increases their susceptibility to gas accumulation, which becomes evident from a steeper slope in the low‐frequency region of the Nyquist plot, reflecting heightened resistance.

Long‐term stability of a single cell using non‐precious metal catalysts was performed at a constant current density of 0.5 A cm^−2^. As shown in Figure 9a,b, the long‐term performance pattern of non‐PGM electrodes is similar to that of PGM electrodes. However, the polarization curves of the MEA with non‐PGM catalysts at initial and after 1000 h of operation differ considerably from those of the MEA with PGM catalysts (Figure 9c). After 1000 h of operation, the cell performance was higher at low current densities, and lower at high current densities than the initial performance. When we operated the cell under identical conditions, a similar degradation of the electrode occurred (Figure S18, Supporting Information). We believe that the degradation evident from the polarization curves after 1000 h of operation resulted from increased mass transport resistance at high current density region due to mechanical damage on the fabricated anode. Variations in the mechanical properties of the electrode can also induce voltage differences, as shown in Figure S18 (Supporting Information). We conducted a performance evaluation through electrode optimization to reduce performance deviation.

## 3 Conclusion

When operating an alkaline electrolyzer with a dense electrolyte membrane, the greatest challenge is ensuring the long‐term stability of the membrane. Low long‐term stability offsets the advantages of the electrolyzer. In this study, we developed a novel method that enhanced the long‐term stability of the membrane. In particular, we focused on improving the stability of PBI by increasing its crystallinity. After synthesizing *p*‐PBI and immediately preparing a membrane, a fe‐*p*‐PBI membrane with high crystallinity was fabricated via a simple biaxial stretching process. This biaxial stretching process is a new technique to effectively fabricate a highly crystalline PBI membrane. The crystallinity of PBI allows the PBI membrane to have high physical strength even if it absorbs a large amount of KOH solution.

The fe‐*p*‐PBI membrane was evaluated for its utility as a membrane by varying the temperature, KOH concentration, and electrocatalysts. The high crystallinity of the fe‐*p*‐PBI membrane maintained its stability even under highly basic conditions. Although fe‐*p*‐PBI was chemically stable, its crystallinity decreased through electrochemical reaction and eventually damaged the membrane. In this study, the durability of the electrolysis cell was tested at 60 °C, as it was thought that polymer deformation would be faster at higher temperatures. Further research is required to investigate the degradation in fe‐*p*‐PBI and the ways to maintain its crystallinity.

## 4 Experimental Section

### Chemicals

3,3′‐Diaminobenzidine, terephthalic acid, isophthalic acid, and polyphosphoric acid (115% phosphoric acid equivalent) were purchased from Sigma–Aldrich and used without further purification. Pt/C (46.9 wt.%, Tanaka, Japan), IrO_2_ (99.99 wt.%, Alfa Aesar), and Nafion perfluorinated ionomer dispersion (5 wt.% dispersion in lower aliphatic alcohols and H_2_O, Sigma–Aldrich) were used to fabricate the MEA of the electrolyzers. Nickel fiber paper (68844, Dioxide Materials, USA, thickness 300 µm) and stainless‐steel fiber paper (68841, Dioxide Materials, USA, thickness 650 µm) were used as the PTL. NiSO_4_•6H_2_O for the fabrication of the Ni‐Fe LDH electrode was purchased from DAEJUNG Chemicals (South Korea).

### Preparation of p‐PBI and m‐PBI Membranes and Fibers

Terephthalic acid (2.3 g, 14 mmol) and 3,3‐diaminobenzidine (3.0 g, 14 mmol) were polymerized in polyphosphoric acid (125 g) under an Ar atmosphere. The polymerization mixture was stirred at 150 °C for 5 h and at 220 °C for 20 h. The viscous polymer solution was poured onto a glass plate and flattened using a doctor blade (blade height: 400 µm) to obtain a uniform thickness. It was then quenched in a water bath, whereupon the membrane detached itself from the glass plate after ≈30 s. Finally, the membrane was soaked in isopropanol to produce the *p*‐PBI membrane. The quenched membrane was stored in isopropanol until use. To fabricate the *p*‐PBI fibers, the polymerization mixture was poured into water to produce fibrous PBI. The fibers were manually extended until just before their breaking point. They were treated with 10 wt.% ammonium hydroxide solution, washed with water and isopropanol several times, and dried under reduced pressure at 100 °C for 30 h. For the preparation of *m*‐PBI membrane and fibers, isophthalic acid was used instead of terephthalic acid. The viscosity of *m*‐PBI was measured using sulfuric acid at 25 °C. The intrinsic viscosity was 104.0 mL g^−1^.

### Biaxially Extended Membrane Fabrication (fe‐p‐PBI)

The quenched membrane (*p*‐PBI) was removed from the storage isopropanol and placed on a glass plate. The four corners of the membrane were then fixed to the glass plate with double clips and the membrane was dried under ambient conditions for 2 days. After the membrane was detached from the glass plate, it was treated with 10 wt.% ammonium hydroxide solution, washed with water, and dried under ambient conditions. The thickness of the membrane was 30 µm.

### sc‐m‐PBI Membrane Fabrication

*m*‐PBI (1.0 g) and LiCl (0.5 g) were dissolved in 49.0 g of dimethylacetamide (DMAc). After filtration to remove undissolved substances, an *m*‐PBI membrane was formed. The thickness of the membrane was adjusted using the doctor blade. The DMAc was removed at 80 °C under reduced pressure. Then the membrane was treated with 10 wt.% ammonium hydroxide solution, washed with water, and dried under ambient conditions. The thickness of the membrane was 30 µm.

### KOH Solution Swelling

The KOH solution swelling, thickness, and area change were determined by measuring the weight, thickness, and area changes, respectively, of the membranes immersed in aqueous KOH solution (20 wt.%) at 80 °C for 24 h. Prior to the test, all the polymers were dried overnight in a vacuum oven at 60 °C. This experiment was repeated three times, and the results were averaged.

(1) $$\mathrm{KOH}\;\mathrm{solution}\;\mathrm{swelling}=\frac{\left(W_{\mathrm{KOH}\;\mathrm{solution}\;\mathrm{wet}}-W_{\mathrm{dry}}\right)}{W_{\mathrm{dry}}}\times \;100$$

(2) $$\mathrm{Thickness}\;\mathrm{change}=\frac{\left(T_{\mathrm{KOH}\;\mathrm{solution}\;\mathrm{wet}}-T_{\mathrm{dry}}\right)}{T_{\mathrm{dry}}}\times \;100$$

(3) $$\mathrm{Area}\;\mathrm{change}\;=\;\frac{\left(A_{\mathrm{KOH}\;\mathrm{solution}\;\mathrm{wet}}\;-\;A_{\mathrm{dry}}\right)\;}{A_{\mathrm{dry}}}\times 100$$

here, W_dry_, T_dry_, and A_dry_ are the weight, thickness, and area, respectively, of the dry membrane, and W_KOH solution wet_, T_KOH solution wet_, and A_KOH solution wet_ are the weight, thickness, and area, respectively, of the membrane after KOH solution swelling.

### Conductivity of fe‐p‐PBI

fe‐*p*‐PBI membrane was immersed in 10 and 20 wt.% KOH solution for 2 days at room temperature. It was then removed and its surface was wiped. Each sample was cut into 5 cm × 1 cm pieces and placed in a temperature and humidity controlled chamber. The IM6 system was used in galvanostatic mode over a frequency range of 100 mHz – 100 kHz. Impedance was measured after the humidity and temperature had equilibrated. Conductivity was calculated using the observed resistance and the following relationship:

(4) $$\mathrm{Proton}\;\mathrm{conductivity}\;\sigma \left(S/\mathrm{cm}\right)=\frac{D}{RA}$$

where D is the distance between two electrodes, R is the measured resistance, and A is the transverse area of the membrane.

### Stability of fe‐p‐PBI in KOH Solution

Several pieces of fe‐*p*‐PBI were immersed in 1 m aqueous KOH solution at 80 °C for 500 h. The fe‐*p*‐PBI pieces were removed from the KOH solution after 24, 200, or 500 h, washed with water, and dried, and their mechanical strength was measured. Each sample was measured three times and the results were averaged. Tensile strength measurements were performed using an H5KT tensile testing instrument (Tinius Olsen; crosshead speed: 100 mm min^−1^) at 25 °C under 50% relative humidity.

### WAXS Analysis

The 2D wide‐angle X‐ray diffraction (WAXS) patterns were recorded on a phosphor image plate (Molecular Dynamicsq) using a Statton camera and monochromatic Cu Kα radiation from a rotating anode X‐ray generator operating at 40 KV and 240 mA. The sample‐to‐membrane distance was calibrated using Si powders. By varying the placement of the samples on the pinhole, the X‐ray beams could be oriented parallel or perpendicular to the membrane surface. The azimuthally averaged 2θ scan was obtained using FIT2D data analysis software [www.esrf.fr/computing/scientific/FIT2D/]

### Electrode Preparation Using PGM Catalysts

A pre‐treatment was performed prior to spray coating on the PTL. First, after 10 min of treatment in NaOH at 90 °C, sonication treatment was performed in 20 wt.% hydrochloric acid for 10 min to remove impurities. It was then sufficiently washed with H_2_O. The catalyst slurries consisted of a catalyst, binder, and solvent (H_2_O and IPA). IrO_2_ and Pt/C were used as the anode and cathode catalysts, respectively. For the anode catalyst slurry, IrO_2_ (13.8 mg) was added as the catalyst, Nafion ionomer dispersion (30.6 mg) as the ionomer, and H_2_O (0.14 g) and IPA (1.4 g) as the solvents. The cathode catalyst slurry was prepared using Pt/C (14.7 mg), Nafion ionomer dispersion (32.6 mg), H_2_O (14.7 mg), and IPA (1.5 g). The slurries were sonicated for 40 min at 40 °C. The catalyst‐coated‐on‐substrate method was used to spray‐coat the anode and cathode catalysts on stainless steel fiber paper and nickel fiber paper substrates, respectively, to a catalyst loading of 2 mg_Ir_ cm^−2^ for the anode and 1 mg_Pt_ cm^−2^ for the cathode.

### Electrode Preparation Using PGM‐Free Catalyst

The Ni‐Fe layered double hydroxide (Ni_‐_Fe LDH) anode was prepared via a corrosion‐engineered growth method. The pretreated Fe foam substrate (2.0 mm, 2,000 g m^−2^, Alantum) was immersed in a 0.185 m Ni(SO_4_)_2_•6H_2_O solution with oxygen sparging through a glass purge tube at 50 °C for 7 h. The Ni_‐_Fe LDH grown on the Fe foam was rinsed with deionized (DI) water and ethanol, and dried in a vacuum desiccator. In addition, a Raney‐type Ni cathode was prepared by depositing Al on Ni foam via a PVD process. Ni foams (1.6 mm, 420 g m^−2^, Alantum) were immersed in a 20 wt.% NaOH solution at 80 °C for 5 min and an 18 wt.% HCl solution at room temperature for 5 min to remove impurities and oxides, respectively. Al was sputtered to a thickness of 10 µm on the pretreated Ni foam using non‐reactive DC‐magnetron sputtering at 300 W, and heat treated at 600 °C for 20 min to produce gamma‐phase Ni‐Al alloys. Subsequently, residual Al was leached with 20 wt.% KOH and 10 wt.% of potassium sodium tartrate solutions at 80 °C, followed by rinsing with water.

### Single Cell Fabrication

The electrolysis cell comprised end plates, bipolar plates (BPs), electrode, membrane, and gasket (anode: Teflon gasket, 560 µm; cathode: Teflon gasket 220 µm). The anode and cathode BPs were stainless steel and graphite, respectively. The flow paths in both the anode and cathode were parallel type. Before the cell tests, the membrane and electrodes (PGM catalyst cell) or membrane only (PGM‐free catalyst cell) were treated with 20 wt.% aqueous KOH solution at room temperature for 2 h. The single cells were assembled under 3.95 Nm of torque.

### Electrochemical Analysis

Single cell tests were performed at 80 °C under ambient pressure on cells with an active area of 6.25 cm^2^. Aqueous KOH solution was delivered into the anode and cathode at a flow rate of 20 mL min^−1^. A pre‐activation process was carried out for 4 h on the membrane and anode and cathode electrodes at the same temperature and KOH concentration that were used as the actual water electrolysis operating conditions. After assembling the cell, a cell activation process was performed for a day in the evaluation system to allow the single cell to reach its peak performance. Electrochemical analysis of the single cells was performed as follows: first, one cycle of linear sweep voltammetry (LSV) was conducted at a sweep rate of 10 mV s^−1^ from 1.35 to 2.0 V. Then, the single cell was activated using chronoamperometry (CA) at 1.55 V for 10 min. This was followed by electrochemical impedance spectroscopy (EIS) measurements and finally, LSV was performed again under the same conditions. EIS was conducted in the frequency range of 50 kHz to 50 mHz at 1.6 and 1.8 V with amplitudes corresponding to 16 and 18 mV, respectively. A long‐term stability test was then conducted at constant current densities of 0.7 A cm^−2^; measurements were recorded once every 10 s. An HCP‐803 (Bio‐Logic, France) and XG 20‐76 (Sorensen Ametek, USA) were used for the single cell and long‐term stability tests, respectively. During the AST, each cycle consisted of five distinct constant‐current density steps, ranging from 0.3 to 1.9 A cm^−2^. The single cell was activated using chronopotentiometry (CP), with each step lasting 3 min. Upon completing a cycle, the next cycle was immediately initiated and this process was repeated for 200 cycles.

### Gas Permeability

FTC 300 (Messkonzept, Germany) sensor was used to measure the volume percentage of hydrogen in oxygen at the anode. The sensor specializes in the measurement of the fractional amount of binary gas mixture, such as H_2_ in O_2_. The electrolytic cell of the anode outline was directly connected to the liquid–gas separator, heat exchanger, and silica gel tank to capture water vapor containing in binary gas mixture. The gas collection time was 10 min at various current densities, with nitrogen purging during the measurement interval. The sensor only detected the percentage of hydrogen volume in oxygen ranging from 0% to 4% up to two decimal places. The degradation of the membrane was indicated once this percentage reached 4%.

## Conflict of Interest

The authors declare no conflict of interest.

## Supporting information



Supporting Information

## Data Availability

The data that support the findings of this study are openly available in Production data at https://doi.org/[doi], reference number 1.
