# Supplementary material for: Long‐Term, Stable Alkaline Electrolysis with a Durable Crystalline Polybenzimidazole Membrane
Source: Small. 2025 Aug 7;21(38):e02810. doi: 10.1002/smll.202502810 (PMC12462583; doi:10.1002/smll.202502810)
Supplement: Supplementary file 1 — Supporting Information [file SMLL-21-e02810-s001.docx]

Supporting Information

Long-Term, Stable Alkaline Electrolysis with a Durable Crystalline Polybenzimidazole Membrane

Tae Kyung Lee^a,b,†^, MinJoong Kim^c,†^, Hyungkyu Cho^d,†^, Seungju Lee^a^, Junghwan Kim^a^, Abu Zafar Al Munsur ^d^, Byeol-Nim Lee^d,^, Ji Eon Chae^e^, Jonghee Han^d^, Hyun Seo Park^a^, Jong Hyun Jang^a^, Sung Jong Yoo^a^, Sae Yane Peak^g^, So Young Lee^a,*^, Hyun-Seok Cho^h,*^, Soo-Young Park^i,*^, Kwang Ho Song^b,*^, Hyoung-Juhn Kim^d,*^

**Figure S1.** Structures of polybenzimidazole derivatives.


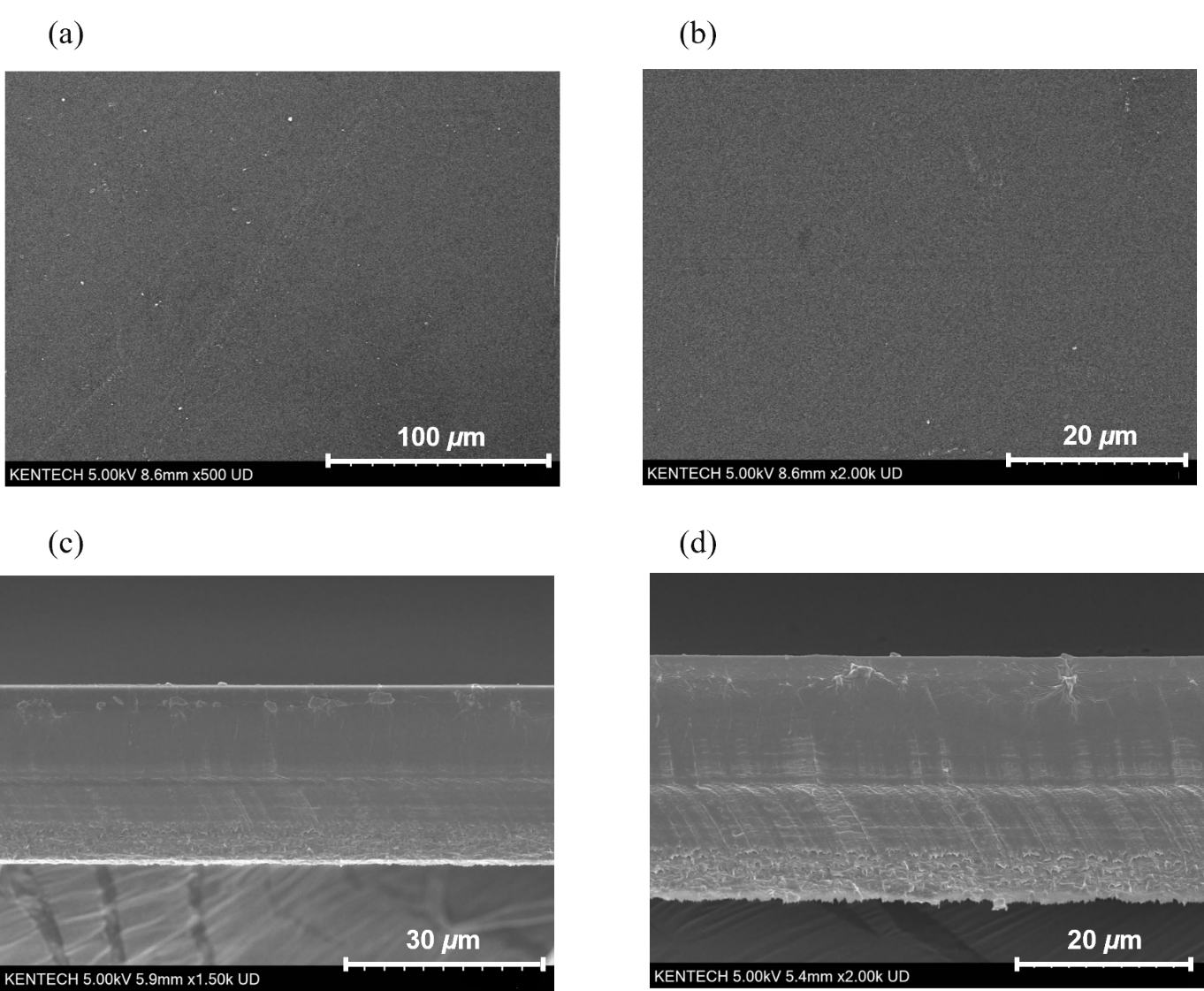


**Figure S2.** Scanning electron micrographs of fe-*p*-PBI (a and b, surface; c and d, cross-sectional area).


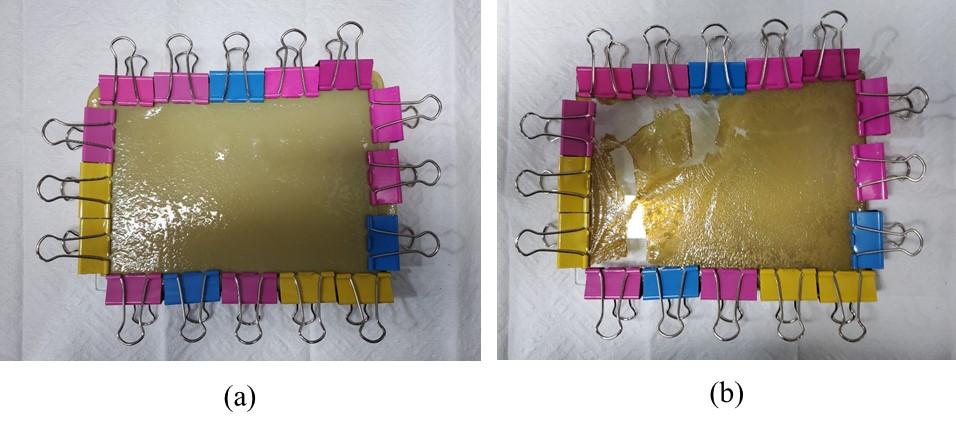


**Figure S3.** Fabrication of fe-*m*-PBI (a) immediately after fixing the film to the glass and (b) after evaporation of isopropanol.


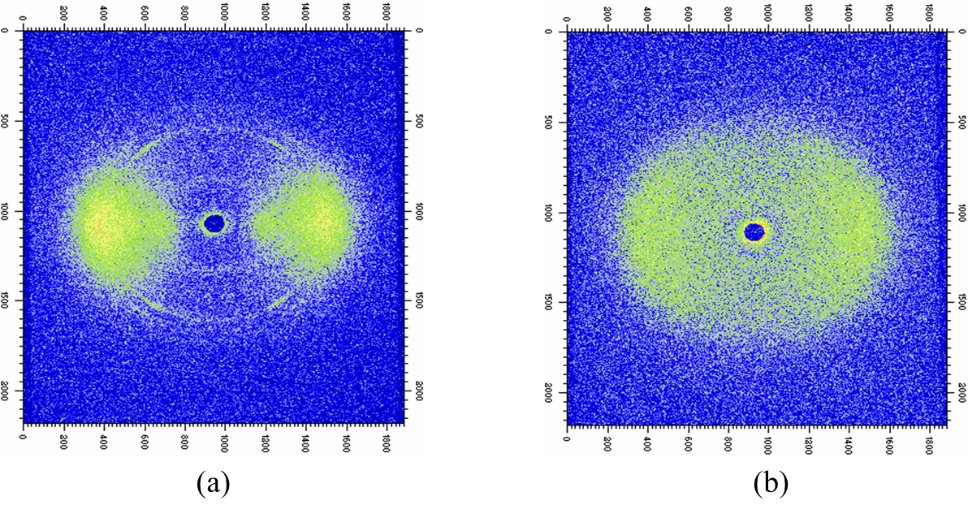


**Figure S4.** Two-dimensional (2D) wide-angle X-ray scattering (WAXS) patterns of (a) *p*-PBI and (b) *m*-PBI fibers.


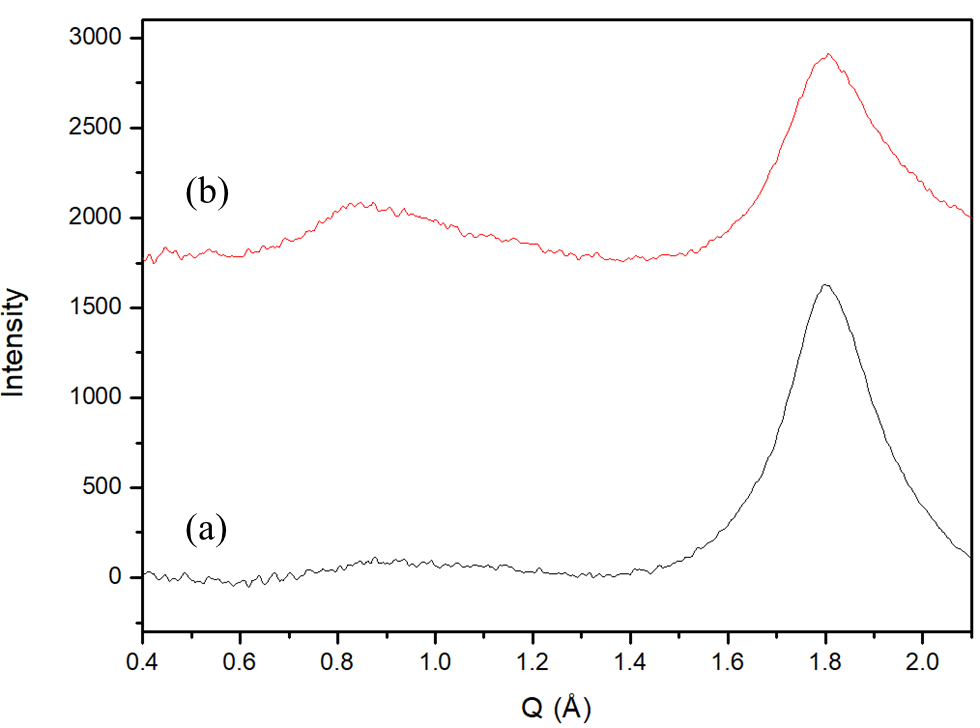


**Figure S5.** WAXS patterns of (a) pristine fe-*p*-PBI and (b) fe-*p*-PBI after treatment in 6 M KOH solution at 80 °C for 250 h.

**
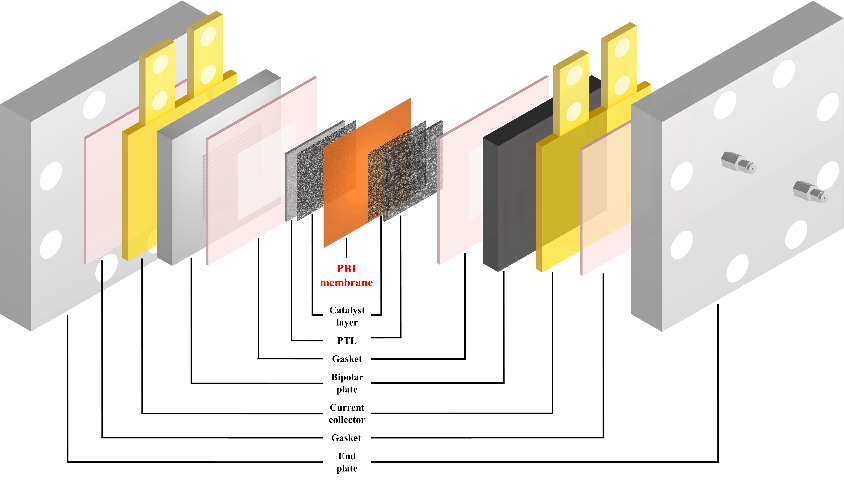
**


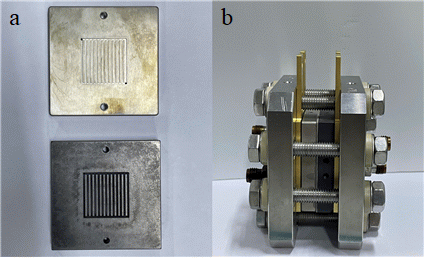


**Figure S6.** Configuration of water electrolyzer. (a) Anode bipolar plate (stainless steel) and cathode bipolar plate (graphite). (b) Single cell.


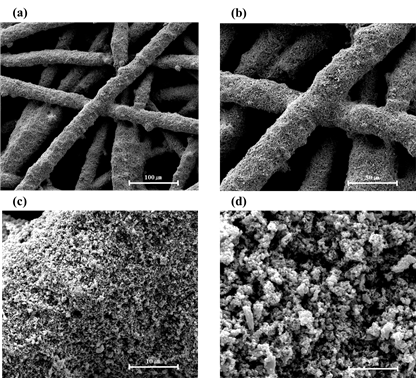


**Figure S7.** Scanning electron micrographs of electrode of IrO_2_ catalyst.


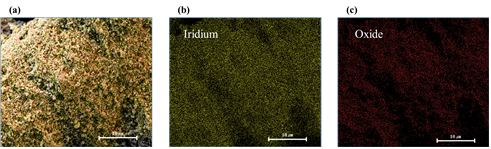


**Figure S8.** Energy dispersive X-Ray spectrometer (EDS) mapping images of electrode of IrO_2_ catalyst.


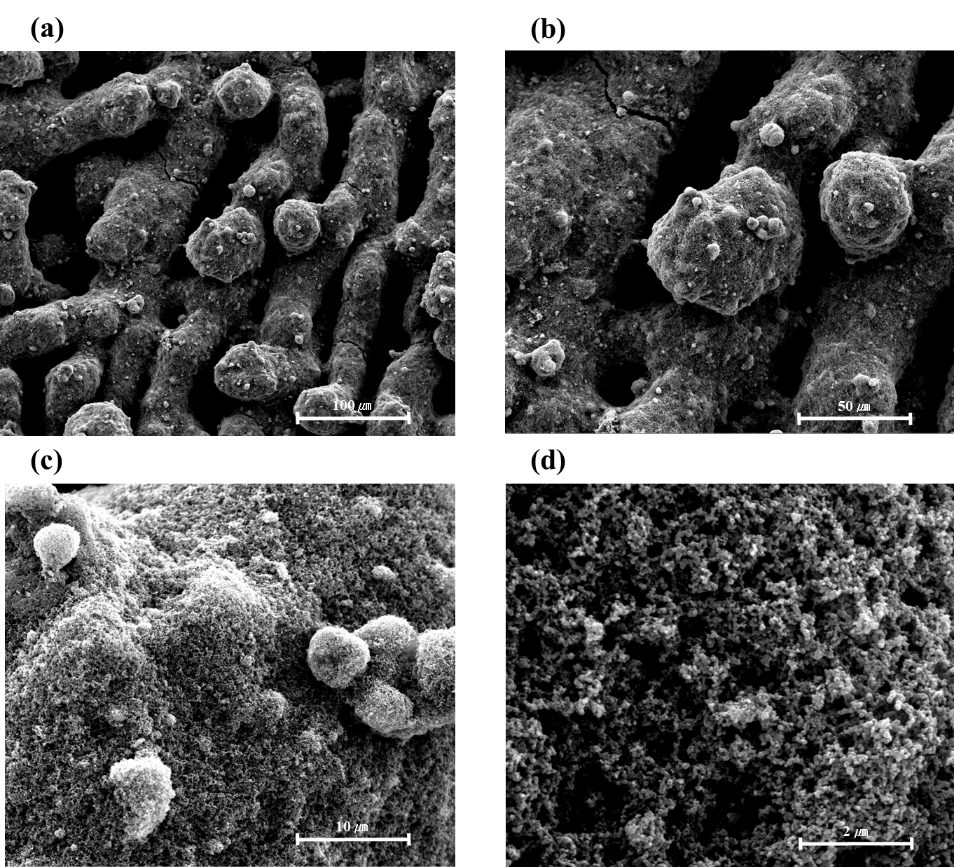


**Figure S9.** Scanning electron micrographs of electrode of Pt/C catalyst.


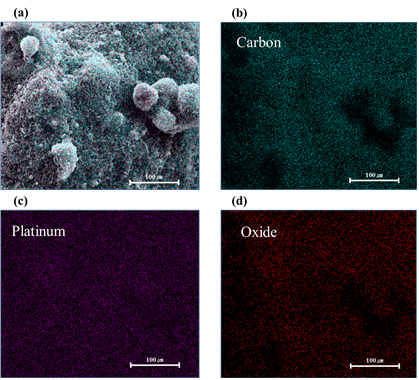


**Figure S10.** EDS mapping images of electrode of Pt/C catalyst.


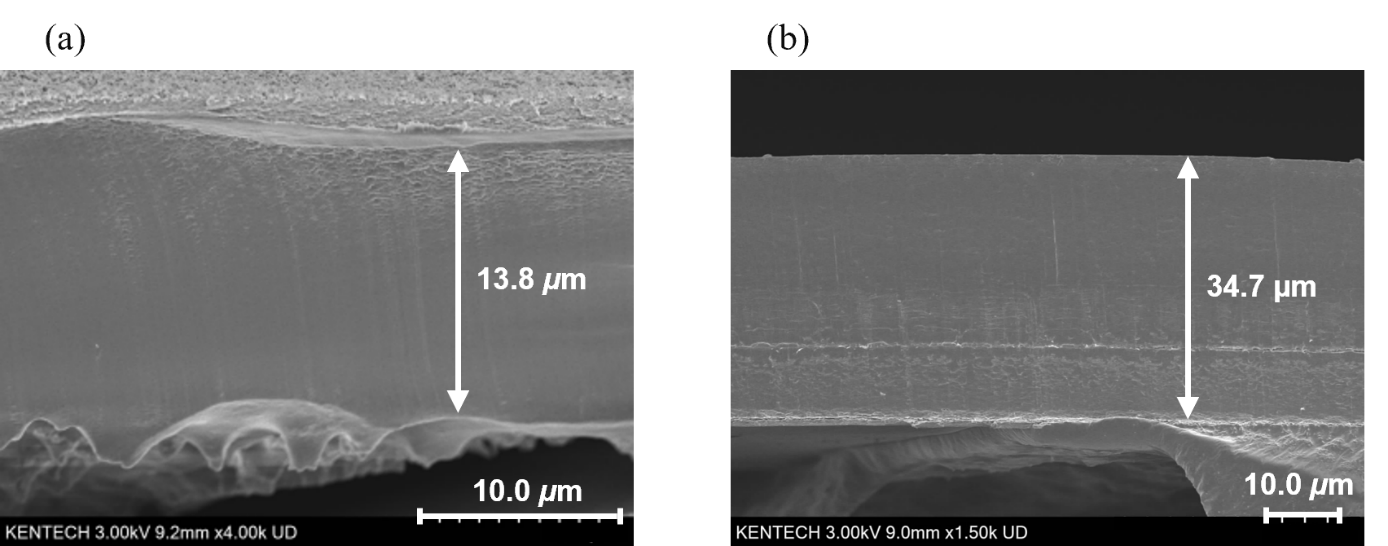


**Figure S11.** Scanning electron micrographs of fe-*p*-PBI cross-section; (a) after 900 h of electrolysis operation and (b) pristine.


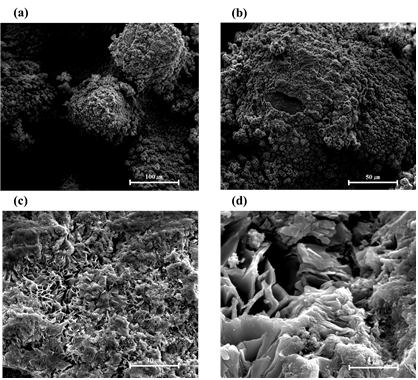


**Figure S12.** Scanning electron micrographs of electrode of Ni-Fe LDH catalyst.


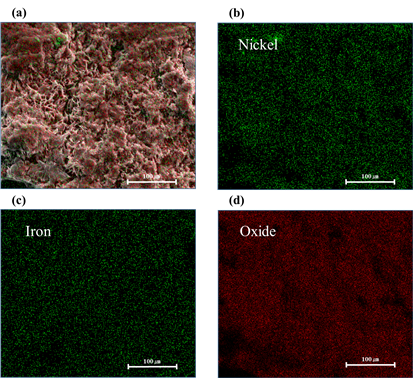


**Figure S13.** EDS mapping images of electrode of Ni-Fe LDH catalyst.


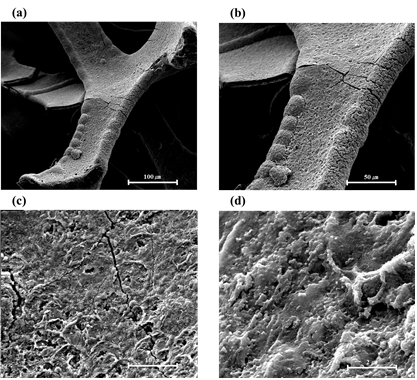


**Figure S14.** Scanning electron micrographs of electrode of Ni-Al catalyst.


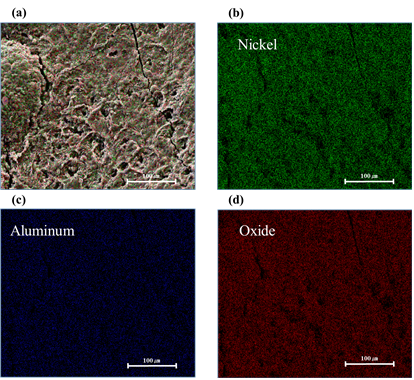


**Figure S15.** EDS mapping images of electrode of Ni-Al catalyst.

**
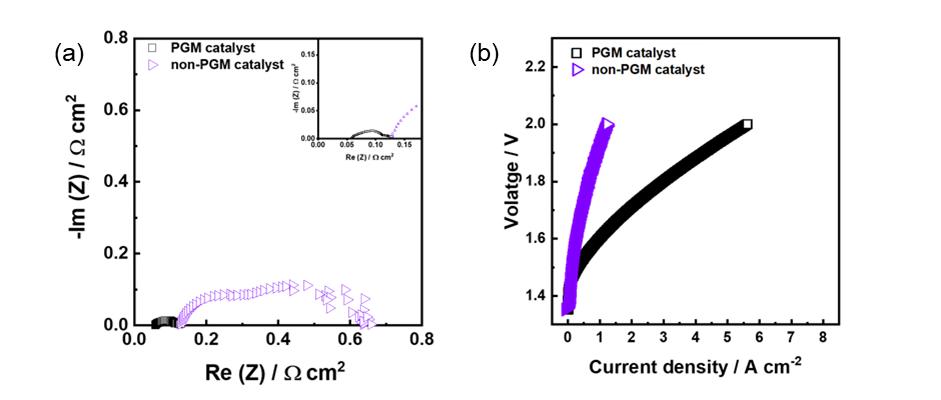
**

**Figure S16.** Electrolysis performance of fe-*p*-PBI. (a) Nyquist plots at 1.6 V. (b) Electrolyzer polarization curves using PGM and non-PGM catalysts (membrane thickness: 30 µm; KOH feed concentration: 20 wt.%; temperature: 80 °C; anode catalyst: IrO_2_ (2 mg_Ir_ cm^-2^) on stainless steel fiber paper (PGM catalyst) and Ni-Fe LDH (non-PGM catalyst); cathode catalyst: Pt/C (1 mg_Pt_ cm^-2^) on Ni fiber paper (PGM catalyst) and Raney nickel (non-PGM catalyst)).

**
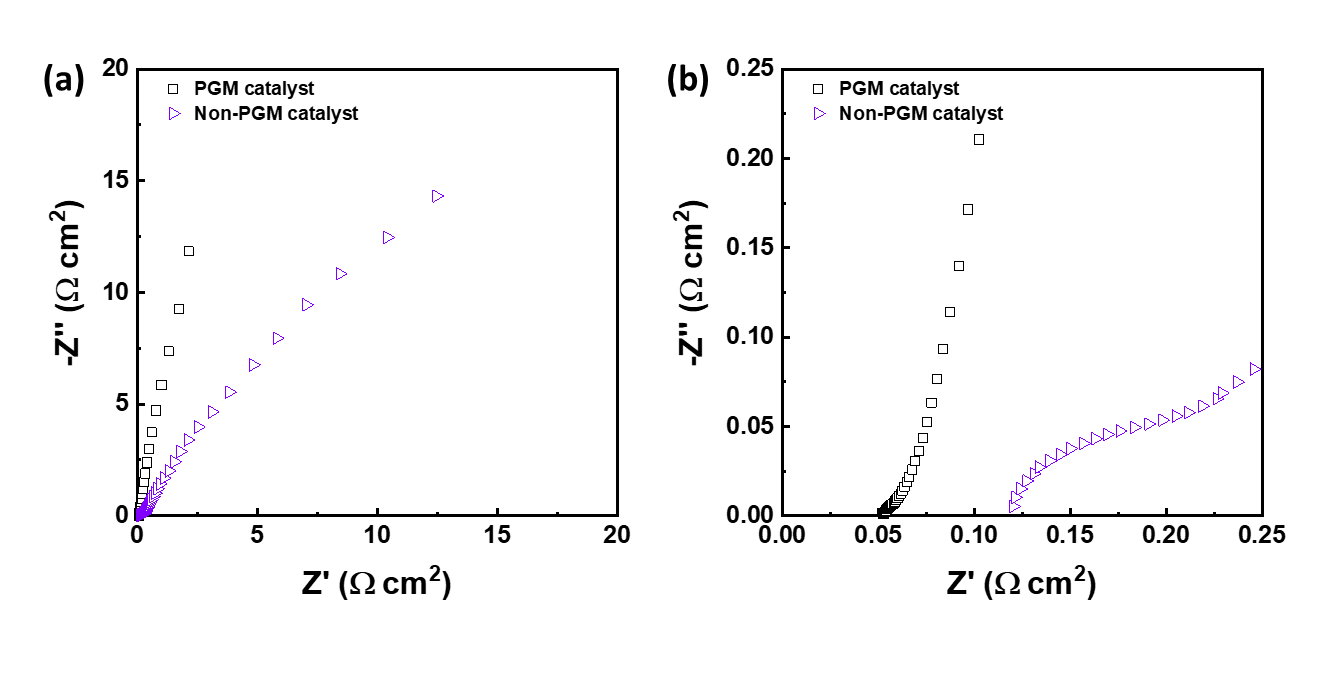
**

**Figure S17.** Electrochemical impedance spectroscopies of PGM- and non-PGM-based electrodes. (a) Nyquist plot at 0 V and (b) Nyquist plot with the modified scale in (a).


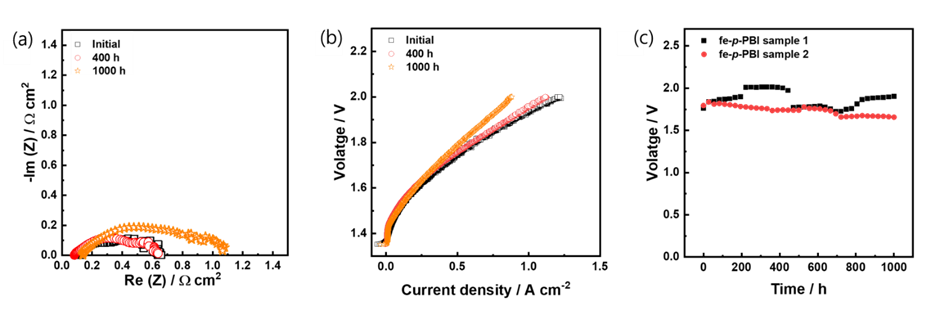


**Figure S18.** Long-term stability of single cell operation at a constant current density of 0.5 A cm^−2^ using fe-*p*-PBI with non-PGM catalysts. (a) Nyquist plots (1.6 V) of initial, 400 h, and 1,000 h operation (sample 1). (b) Polarization curves of initial, 400 h, and 1,000 h operation (sample 1). (c) Long-term stability during 1,000 h operation of two fe-*p*-PBI membrane samples (samples 1 and 2). Membrane thickness: 30 µm; KOH feed concentration: 20 wt.%; temperature: 80 °C; anode catalyst: Ni-Fe LDH; cathode catalyst: Ni-Al.

**Figure S19.** ATR spectra of fe-*p*-PBI and sc-*m*-PBI.

**Table S1.** Conductivity of fe-*p*-PBI at 60 and 80 ℃ after immersion in different KOH solutions

| Temperature (°C) | Conductivity (mS cm^-1^) | |
| --- | --- | --- |
|  | Treatment in 10 wt.% KOH sol. | Treatment in 20 wt.% KOH sol. |
| 60 | 67.6 | 87.4 |
| 80 | 72.7 | 103.7 |

**Table S2.** Mechanical properties of fe-*p*-PBI as a function of immersion time in KOH solution

| Time  (in 80 °C, 1 M KOH sol.) | Mechanical properties | |
| --- | --- | --- |
|  | Tensile strength (MPa) | Elongation (%) |
| 24 h | 1,120 | 14.1 |
| 200 h | 1,020 | 15.7 |
| 500 h | 1,160 | 14.5 |
